# Increased Belowground Carbon Allocation Reduces Soil Carbon Losses Under Long‐Term Warming

**DOI:** 10.1111/gcb.70561

**Published:** 2025-10-16

**Authors:** Andreas Schindlbacher, Steve Kwatcho Kengdo, Jakob Heinzle, Ye Tian, Mathias Mayer, Josef Gadermaier, Chupei Shi, Caro Urbina Malo, Xiaofei Liu, Erich Inselsbacher, Robert Jandl, Carlos A. Sierra, Wolfgang Wanek, Werner Borken

**Affiliations:** ^1^ Department of Forest Ecology and Soils Federal Research and Training Centre for Forests, Natural Hazards and Landscape—BFW Vienna Austria; ^2^ Department of Soil Ecology Bayreuth Center of Ecology and Environmental Research (Bayceer), University of Bayreuth Bayreuth Germany; ^3^ Lawrence Berkeley National Laboratory Climate & Ecosystems—Climate Dept Berkeley USA; ^4^ Division of Terrestrial Ecosystem Research, Department of Microbiology and Ecosystem Science, Center of Microbiology and Environmental Systems Science University of Vienna Vienna Austria; ^5^ Department of Soil and Environment Swedish University of Agricultural Sciences (SLU) Uppsala Sweden; ^6^ Institute of Forest Ecology, Department of Ecosystem Management, Climate and Biodiversity BOKU University Vienna Austria; ^7^ Forest Soils and Biogeochemistry Swiss Federal Institute for Forest, Snow and Landscape Research (WSL) Birmensdorf Switzerland; ^8^ Department of Ecosystem and Landscape Dynamics, Institute for Biodiversity and Ecosystem Dynamics University of Amsterdam Amsterdam the Netherlands; ^9^ Institute of Earth System Sciences, Faculty of Natural Sciences Leibniz University Hannover Hannover Germany; ^10^ Key Laboratory of Humid Subtropical Eco‐Geographical Process of Ministry of Education, School of Geographical Sciences Fujian Normal University Fuzhou China; ^11^ Institute of Soil Research, Department of Ecosystem Management, Climate and Biodiversity BOKU University Vienna Austria; ^12^ Department of Biogeochemical Processes Max Planck Institute for Biogeochemistry Jena Germany

**Keywords:** radiocarbon, root respiration, roots, soil CO_2_ efflux, soil organic carbon, warming

## Abstract

The response of the carbon cycle in forests to global warming could lead to a positive climate feedback if warming accelerates the mineralization of soil organic carbon (SOC), thereby causing net emissions of CO_2_ into the atmosphere. In Europe, carbon‐rich alpine forest soils could be particularly affected by global warming, as a greater rise in temperature is expected in this region than the global average. Here we show that nearly two decades of experimental soil warming (+4°C during the snow‐free seasons) in a mountain forest in the Northern Limestone Alps significantly (~13% per 1°C warming) and persistently (no change in response over 18 years) increased soil CO_2_ effluxes. The SOC stocks in the warmed plots decreased compared to controls, yet non‐significantly, and quantitatively much less than the surplus carbon outflux from warmed soil suggests. We attribute the increase in soil CO_2_ efflux primarily to stimulation of root respiration, which was most sensitive to long‐term warming. Furthermore, increased root production, faster fine root turnover, and increased root exudation likely not only facilitated autotrophic respiration but also replenished the SOC pool. The radiocarbon age of SOC indicates a rejuvenation of SOC likely by increased input of root carbon into the lower topsoil. Overall, our findings suggest that increased C allocation into the rhizosphere can at least partially compensate for the C loss through increased SOC mineralization with rising temperatures over many years.

## Graphical abstract

In this synthesis, we combine new findings with previously published results to evaluate the response of an alpine forest soil to long‐term warming in Achenkirch, Austria. The main focus was on investigating the soil carbon cycle, in particular whether long‐term warming leads to losses of soil organic carbon. To this end, soil respiration was measured over a period of 18 years. Studies on the soil carbon cycle also include microbial biomass, substrate utilization efficiency, carbon utilization efficiency, fine root biomass, fine root turnover, organic carbon concentration, organic carbon stocks, dissolved organic carbon, and radiocarbon‐based modeling of the age distribution of soil organic carbon. Interactions between the carbon, nitrogen, and phosphorus cycle were also part of the investigations.

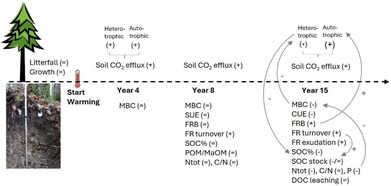

## 1 Introduction

Forest soils store about 730 Pg organic carbon (C) or 40% of total global SOC (Shi et al. 2020). This is equivalent to about 80% of the amount of C in the atmosphere (Masson‐Delmotte et al. 2021). Undisturbed forest soils overall act as a sink for atmospheric carbon dioxide (CO_2_), but rising temperatures could affect the relationship between C inputs by plants and C release through the mineralization of SOC, and thus the C balance between soils and the atmosphere. Global warming may be exacerbated if soils lose organic carbon through increasing mineralization and release of CO_2_ into the atmosphere. However, the likelihood of positive soil carbon‐climate feedback remains uncertain given the paucity of long‐term studies on the response of soils to warming. Field manipulation experiments are one way of simulating rising soil temperatures and tracking changes in C cycling processes, C fluxes, and C stocks under real‐world conditions.

A recent meta‐analysis (Bai et al. 2023) showed no clear change in SOC storage across 47 warming experiments in forests, but there appeared a general trend towards decreasing soil C stocks with increasing warming duration, emphasizing the importance of long‐term experiments. Statistically verified changes in SOC stocks are difficult to measure within few years of experimental warming, due to high stone content, heterogeneous distribution of organic matter, and variation in soil density causing high small‐scale variability of SOC stocks in forest soils (Smith 2004).

As an alternative to measuring SOC stocks directly, C cycle processes are more sensitive indicators of climate change, as they indicate shifts in SOC source and sink processes. The release of CO_2_ via mineralization of litter and soil organic matter (heterotrophic respiration) is the most important SOC loss process. Another CO_2_ source in the soil is autotrophic respiration through roots and associated mycorrhiza.

The contribution of autotrophic and heterotrophic respiration to soil CO_2_ efflux is not constant and can shift with soil warming. Heterotrophic soil respiration shows an inherent positive relationship with soil temperature (Davidson and Janssens 2006), but the temperature sensitivity is controlled by changes in the amount and quality of soil organic matter, and by the size of the soil microbial community and its efficiency in utilizing C for growth (carbon use efficiency, CUE) (Walker et al. 2018). Autotrophic respiration is also temperature sensitive and can change by soil warming in the long term, due to physiological adaptations and changing root and mycorrhizal biomass (Boone et al. 1998; Burton et al. 2008; Jarvi and Burton 2020). Another SOC loss is leaching of dissolved organic carbon (DOC), although the response to warming is likely to be small (Fröberg et al. 2013).

Belowground C inputs through the tree root system represent a major flux to forest soils but are difficult to quantify. The root system of trees can respond to warming by adapting productivity and turnover of fine roots and of mycorrhizal fungi (Wang et al. 2021). The exudation of low‐molecular C‐compounds by fine roots and mycorrhizal hyphae also responds sensitively to soil warming (Yin et al. 2013). It remains unclear whether changing belowground allocation under warming can compensate for increased C losses from soil.

Radiocarbon measurements of SOC are a powerful tool to detect long‐term shifts in SOC pools by changing environmental conditions, which are often not detectable by classical surveys of SOC stocks (Trumbore 2000). Modeling the radiocarbon age and transient time distributions provides additional information about losses or gains of younger and older SOC (Sierra et al. 2017). The few studies using radiocarbon data indicate that soil warming may result in losses (Vaughn and Torn 2019), gains (Finzi et al. 2020), or no changes (Briones et al. 2021) in SOC pools. However, the potential of SOC losses is not well understood and may vary among climate zones, land use, soil characteristics, and other factors.

Here, we synthesize C flux, stock, and isotope data from the Achenkirch long‐term soil warming experiment which is running for almost two decades (since 2005) in a temperate mountain forest in the Austrian Limestone Alps. Alpine soils on calcareous bedrock are typically shallow, contain high amounts of SOC (9%–17%) (Baritz et al. 2010; Kobler et al. 2019; Wiesmeier et al. 2014), have near‐neutral pH, and exhibit high carbonate contents. Repeated soil inventories indicated that these soils may be particularly prone to C losses under warming (Prietzel et al. 2016). While there was emphasis on potential adaptations of the soil microbial community and on changes in SOC quality throughout the Achenkirch soil warming experiment (Schindlbacher et al. 2011; Schnecker et al. 2016), we focused on fine root (Kwatcho Kengdo et al. 2022) and nutrient (Tian, Shi, et al. 2023) dynamics during the final years. Soil CO_2_ fluxes were measured regularly throughout the whole warming experiment. Here, we establish for the first time a relationship between CO_2_ fluxes and SOC contents and stocks in the control and long‐term warmed plots, together with an assessment of radiocarbon signatures and modeling of the age distribution of SOC in the entire soil profiles. The soil warming experiment was established to test if warming leads to SOC losses to the atmosphere. We initially hypothesized that soil warming increases the mineralization of SOC and that the increase in microbial respiration leads to a substantial decrease in SOC stocks over time. We further hypothesized that younger, unstable C is preferentially decomposed under warming, leading to a higher average carbon age in warmed soils and to a decline of the respiratory response to soil warming over time.

## 2 Materials and Methods

### 2.1 Study Site and Soil

The study site is located at 910 m a.s.l. on a gentle north–north‐east exposed slope of a mountain in the Northern Limestone Alps, Achenkirch, Austria (47°34′50″ N; 11°38′21″ E). The ~125‐year‐old forest is composed of Norway spruce ( *Picea abies* , ~80%) and European beech ( *Fagus sylvatica* , ~20%), which also dominate the undergrowth. Soils show a heterogeneous mosaic of Chromic Cambisols and shallow Rendzic Leptosols (WRB 2006), with high carbonate content and near neutral soil pH. Root density is highest in the O‐ and A‐horizons. O‐layer depths (0.5–4 cm) as well as mineral soil layer depths (10–40 cm) are highly heterogeneous depending on microtopography. The climatic conditions at the study site are cool and humid, illustrated by a mean annual air temperature and precipitation of 7.0°C and 1493 mm, respectively (1988–2017, ZAMG weather station: Achenkirch). Nitrogen (N) deposition in precipitation at the site ranged between 7 and 12 kg N ha^−1^ year^−1^ during the past two decades (Jandl et al. 2012).

### 2.2 Soil Warming

The soil warming experiment was designed as a paired plot design. Twelve 2 m × 2 m plots were grouped into six blocks (pairs), established in 2004 (*n* = 3 blocks, start of warming May 2005) and 2007 (*n* = 3 blocks, start of warming April 2008). Two treatments, ambient soil temperature (control) and increased soil temperature by 4°C (warmed), were assigned to the two plots of each block. Soil heating cables (Etherma, Salzburg, Austria) were installed at a mineral soil depth of ~3 cm, with ~7.5 cm spacing between cables in the warming and the control plots (the control plots were not heated but cables were installed to control for soil disturbance). Soil temperature in the warmed plots was maintained at a constant 4°C higher than in the paired control plots throughout the snow‐free period (April/May–November/December). In 2008 and 2009, three blocks received an additional short‐term drought treatment by roofing both control and warming plots for 3 weeks in July/August (Schindlbacher et al. 2012). Soil warming was suspended in 2014 to test for potential adaptation effects to warming. Soil temperatures and soil moisture were recorded every half hour by permanently installed sensors at depths of 5 and 15 cm. In addition, soil temperatures at 5 cm soil depth were measured with a handheld probe during each CO_2_ flux measurement campaign. For the last 4 years (2019–2022), soil moisture was measured manually at 0–7.5 cm soil depth with a FieldScout soil moisture meter (TDR 100, Spectrum Technologies Inc., USA). Air temperature, precipitation, and relative humidity were measured in hourly intervals at a climate station (operated by Hydrographischer Dienst Tirol) located about 100 m distance from the experimental plots. Vapor pressure deficit (hPa) was derived from air temperature and relative humidity measurements. Further details of the experimental setup and the instrumentation can be found elsewhere (Heinzle, Kitzler, et al. 2023; Schindlbacher et al. 2012).

### 2.3 Soil and Root CO_2_ Efflux

Soil CO_2_ fluxes were measured biweekly to monthly from April 2005 to December 2022. During the snow‐free seasons, soil respiration was measured from three randomly distributed chambers (20 cm diameter, 10 cm height) on each plot. CO_2_ concentrations were measured using EGM4 and EGM5 infrared gas analyzers (PP‐Systems, Amesbury). The soil respiration measurements of all chambers took almost 4 h. To ensure a consistent measurement protocol, we started the CO_2_ flux measurements between 9 and 10 am. The order of CO_2_ flux measurements was randomised, but a measurement in a control plot was always followed by a measurement in the paired warming plot. Soil CO_2_ fluxes were calculated:

$$\begin{array}{cc} {\mathrm{RCO}}_{2}= & \Delta C/\Delta t\;\times \;273.15/\left(T_{\mathrm{air}}+273.15\right)\;\\ & \times p/1000\;\times \;22.41/1000\;\times \;V/A, \end{array}$$

where RCO_2_ is the CO_2_ flux rate from the soil surface (μmol m^−2^ s^−1^), Δ*C*/Δ*t* is the concentration change (ppm) over time (120 s), *T*_air_ is the air temperature (°C), *p* is the atmospheric pressure (Pa), M the molecular weight (g mol^−1^), 22.41 is the molar volume of an ideal gas at standard temperature and pressure (1 mol^−1^), *V* is the chamber volume (m^3^), and *A* is the chamber area (m^2^). The term (*T*_air_ + 273.15) is used for the conversion of air temperature from degree Celsius to degree Kelvin. During snow cover, soil CO_2_ fluxes were estimated by measuring within snow‐cover CO_2_ concentration profiles (Schindlbacher et al. 2007). In the spring of each treatment year, we measured soil CO_2_ fluxes before turning on the warming system and repeated the measurement the day after (+4°C reached) to test for the short‐term response to warming across the study years.

To estimate seasonal and annual CO_2_ efflux from the control and warming plots, we followed two approaches. (1) We simply linearly interpolated between consecutive CO_2_ measurement dates and summed to get cumulative annual efflux rates. However, this approach can be problematic if the CO_2_ measurements are infrequent. Therefore, we additionally (2) modeled daily soil CO_2_ fluxes for each plot, using the plot‐specific relationship between soil temperature and soil CO_2_ efflux, which were then aggregated to sums of the corresponding study years. A Gaussian model provided the best fit between soil CO_2_ efflux rates as a function of soil temperature:

$$R\left(T\right)=Re^{\mathrm{aT}+bT^{2}}$$

where *R*(*T*) is the measured soil CO_2_ efflux at a soil temperature *T* at 5 cm soil depth. Model parameters (*R*, *a*, *b*) were obtained individually for each calendar year by fitting the function (SigmaPlot 14.0, dynamic curve fitting) to the plot‐specific CO_2_ fluxes and soil temperatures recorded during the flux measurements. We applied the model to a three‐year data‐window, including the year under consideration, the previous year and the following year. The plot specific model parameters obtained for the corresponding calendar year were then, in combination with the specific mean daily soil temperature, used to calculate plot specific daily soil CO_2_ efflux rates. Daily plot efflux estimates were summed up to annual cumulative CO_2_ efflux values. Soil temperature explained a large part of the temporal variation of the CO_2_ efflux in the Gaussian model (mean *R* ^2^ = 0.90 ± 0.03 across all plots and years). Integration of a soil moisture term did not result in further improvement of the model predictability.

Respiration from tree fine roots was measured in June and September 2022. Tree fine roots (< 2 mm diameter) were excavated from warmed and control plots (from five locations within each plot at 0–5 cm soil depth). The sampled roots were pooled to a single sample per plot and cleaned by shaking them free of adhering soil and using a brush and tweezer to remove further litter and soil particles. Cleaned fine roots (3–5 g fresh weight) were placed in a 325 mL glass incubation chamber, which was then wrapped in aluminum foil to achieve complete darkness. The incubation chamber was placed in a water bath with the exact temperature of the corresponding soil during root sampling. Respiration measurements started after a 15‐min equilibration period. During the equilibration period, the incubation chamber was flushed with air from a second, larger chamber in the water bath, which was filled with air with a similar CO_2_ concentration as the topsoil of the corresponding plot (the actual CO_2_ concentration in the soil air at 5 cm depth was measured prior to root sampling). After equilibration, the increase in CO_2_ concentration in the root incubation chamber was monitored for 30 min with an EGM5 infrared gas analyser (PP‐Systems, Amesbury) in a closed dynamic system (air flow rate 100 mL min^−1^). We applied a linear fit (all *R* ^2^ > 0.996) to the increase in CO_2_ concentration during minutes 5 and 15 for the actual flux calculation. After measuring fluxes at the individual plot temperatures, root respiration from all plots was measured at a single reference incubation temperature (17°C) to test for adaptation effects to long‐term warming. Finally, roots were then taken to the laboratory, washed, and dried at 80°C for 24 h to calculate the flux per g fine root dry mass.

### 2.4 Soil Organic Carbon Concentrations and Stocks

Soil organic carbon concentrations and stocks from all plots were determined in the year 2019. For measuring SOC contents, soil was sampled three times (spring: 2nd May, summer: 6th August, and autumn: 15th October 2019). In each plot, 6–7 soil cores were taken per season using a stainless steel auger (diameter 2.5 cm); soil samples were separated into 0–10 cm and 10–20 cm depth increments. Soil samples were pooled per plot, soil depth, and season into 72 soil samples that were analysed for SOC, soil total N (TN) concentrations, and other microbial parameters (Tian, Schindlbacher, et al. 2023). Pre‐warming SOC concentration assessments were not available, but we recovered deep‐frozen topsoil sampled at ~5 cm soil depth from all 12 plots in 2008–2009 (Kuffner et al. 2012). These samples were analysed for SOC and TN using the same protocol as for the 2019 samples. For determining bulk density, we opened two small soil pits at each plot and carefully inserted stainless steel cylinders horizontally in the centers of the 0–10 and 10–20 cm depth layers. Samples were oven dried at 105°C for 24 h, and the mass of the soil and all stones was determined together with their volumes. Bulk density of the fine soil was calculated as:

$${\mathrm{BD}}_{\text{fine soil}}=\frac{{\text{mass}}_{\text{sample}}-{\text{mass}}_{\text{rock fragments}}}{{\text{volume}}_{\text{sample}}-{\text{volume}}_{\text{rock fragments}}}$$

where ${\mathrm{BD}}_{\text{fine soil}}$ is the bulk density of the fine soil, ${\text{mass}}_{\text{sample}}$ is the total mass of the sample, ${\text{volume}}_{\text{sample}}$ is the total volume of the sample, ${\text{mass}}_{\text{rock fragments}}$ is the mass of the rock fragments and ${\text{volume}}_{\text{rock fragments}}$ is the volume of the rock fragments (measured by H_2_O displacement). The rock fragment content in all samples from the 0 to 10 cm soil layer was minimal (< 1 Vol%), whereas rock fragments ranged from 0% to 24% of the volume in the 10–20 cm layer samples. Soil organic C stocks were calculated for each plot and soil layer as:

$${\text{SOCstock}}_{i}={\bar{\text{SOCconc}}}_{\text{fine soil}}\times {\bar{\mathrm{BD}}}_{\text{fine soil}}\times {\text{depth}}_{i}$$

where ${\text{SOCstock}}_{i}$ is the plot specific SOC stock of the investigated layer (g cm^−2^), ${\bar{\text{SOCconc}}}_{\text{fine soil}}$ is the mean SOC concentration in the fine soil (%) of the plot, ${\bar{\mathrm{BD}}}_{\text{fine soil}}$ is the mean bulk density of the fine soil in the plot (g cm^−3^), and ${\text{depth}}_{i}$ is the depth of the respective soil layer (cm).

The average bulk density of the mineral soil was ~20% higher in the warmed plots, but the difference to the controls was not significant. It is likely that long‐term warming led to soil compaction, for example, as a matter of aggregate destabilization (Poeplau et al. 2020). To account for soil compaction, we applied a layer‐thickness correction in warmed plots according to Verbrigghe et al. (2022), assuming equal soil bulk densities in both treatments at the start of the experiment. For the topsoil (0–10 cm depth) from the warmed plots, we calculated a corrected layer thickness corresponding to the respective bulk density in the control plots. Using the ratio of corrected and uncorrected layer thickness, we calculated a corrected SOC stock for the warmed topsoil. For the warmed subsoil (10–20 cm depth), we corrected the thickness in the same way as for topsoil but subtracted the surplus thickness of the above topsoil. The equations for the thickness‐dependent correction of soil mass and the calculation of layer‐specific SOC stocks are provided in detail in “Appendix B: Supplementary, B1 and B2” in Verbrigghe et al. (2022).

### 2.5 Aboveground Litterfall

Litterfall was monitored at the Achenkirch site since 2007 using litter traps with an area of 0.5 m^2^ each, systematically distributed to cover the entire site variability. All litter traps were placed 1.5 m above the forest floor. The litter within the traps was collected every second month, except during the snow season from December to March, for which the accumulated litter was collected after snowmelt. Aboveground litter samples were oven‐dried at 80°C and weighed. Samples were stored until radiocarbon analysis, and aboveground C input by litterfall for each year was calculated, assuming a constant C concentration of 50% of dry matter.

### 2.6 Radiocarbon Analysis of Fine Roots, Aboveground Litter, and SOC

Fine roots (< 2 mm) and mineral soil were taken at 0–10 cm and 10–20 cm depth from six plots in 2012 and from twelve plots in 2019. See Kwatcho Kengdo et al. (2022) for details on sampling and processing of fine roots. Aliquots of dried live fine roots and aboveground litter (see above) from both years were pretreated with an acid–base–acid treatment to remove non‐structural carbohydrates and organic contaminants that may post‐date aboveground litter and fine root formation (Gaudinski et al. 2001). Mineral soil samples were prepared by removing stones, roots, litter, and macrofauna, sieved (2 mm), and then frozen at −24°C for further analyses. Approximately 5 g of frozen soil from each soil sample was equally decarbonated with 2 M HCl at 25°C for a few weeks. The carbon contained in fine roots, aboveground litter, and soil samples was converted into graphite using the sealed‐tube zinc reduction method described by Xu et al. (2007). Graphite samples were analyzed at the Keck‐CCAMS Facility at the University of California, Irvine, USA, where the radiocarbon signature of all samples was measured using accelerator mass spectrometry (AMS, NEC 0.5MV 1.5SDH‐2 Pelletron, National Electrostatics Corporation, Middleton, Wisconsin, USA) (Southon et al. 2004). Radiocarbon data are expressed as Δ^14^C, which is the per mil deviation from the ^14^C/^12^C ratio of oxalic acid standard in 1950. The sample ^14^C/^12^C ratio has been corrected to a *δ* ^13^C value of −25‰ to account for any mass‐dependent fractionation effects.

### 2.7 Modeling of SOC Age and Transit Times of CO_2_

A radiocarbon curve integrating the pre‐ and post‐bomb period was constructed for atmospheric CO_2_ by interpolating the Intcal13 dataset (northern hemisphere atmospheric ∆^14^C for years < 1986) (Reimer et al. 2013) and ^14^C records of atmospheric CO_2_ measured at the Hohenpeißenberg (2015–2020) (Kubistin et al. 2021) and the Schauinsland stations (1986 to 2016) located in the south of Germany (Levin and Hammer 2017).

**FIGURE 1:**
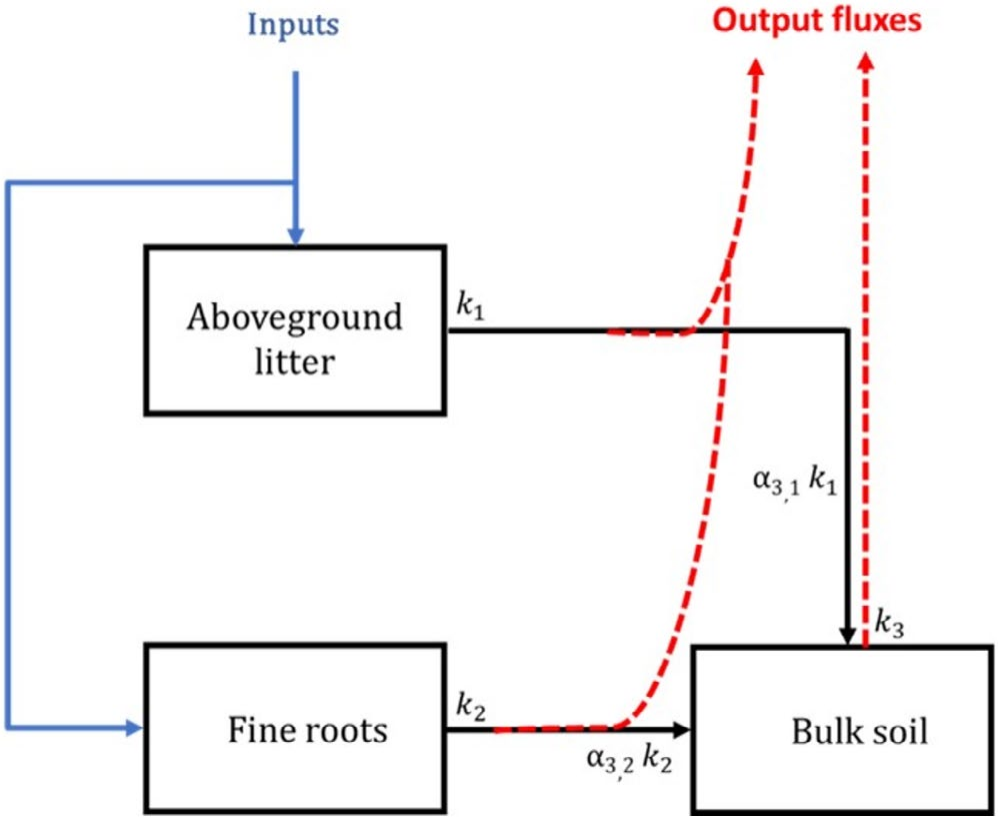
Structure of the model used to estimate radiocarbon distributions at 0–10 cm and 10–20 cm soil depths. $k_{1}$, $k_{2}$, and $k_{3}$ are the decay rates (year^−1^) in aboveground litter, fine roots, and bulk SOC, respectively. $a_{3,1}$ and $a_{3,2}$ describe the proportion of C transferred from aboveground litter to bulk SOC on the one hand and from fine roots to bulk SOC on the other.

We used a steady‐state compartment model using the *SoilR* package, version 1.2.105 (Sierra et al. 2014, 2012) to estimate the age and transit time of C in control and warming treatments. This model considered two soil depth separately (0–10 cm and 10–20 cm), assuming no relevant C transfer occurs between them (Figure 1). At each soil depth, the model comprises three compartments: aboveground litter, fine roots, and bulk SOM. Organic carbon enters the system as aboveground litter (compartment 1) and fine roots (compartment 2). The C in those two compartments is subject to exponential decay, with decay rates $k_{1}$ and $k_{2}$, respectively (Figure 1). Following that decay, a fraction of the decomposed litter in both compartments is lost (output flux), the remaining is transferred to the bulk SOM, and the transfer coefficients $a_{3,1}$ and $a_{3,2}$ describe the rate of this flux, respectively. The C in the bulk SOM is also subject to decay, represented by $k_{3}$. A fraction of that C is also lost, and the remaining accumulates as SOC. Dissolved organic carbon (DOC) input by throughfall was not considered in the model, as it is assumed to be rapidly mineralized by soil microbes.

The steady‐state compartment model is described with the following equation:

$$\frac{d_{C}\left(t\right)}{d_{t}}=I+B\left(t\right)\;C\left(t\right)$$

where I is a constant vector that describes the inputs of C to each compartment in the system at a time t; B is a matrix of decomposition and transfer rates within the system, and C is a vector of C stocks in each compartment. Following this general formulation, we represented the model at 0–10 cm and 10–20 cm soil depth with:

$$\left(\begin{array}{c} \mathrm{AG}\;\text{litter}\\ \text{Fine roots}\\ \text{Bulk}\;\mathrm{SOM} \end{array}\right)=\left(\begin{array}{c} \text{Cinput}\\ \text{Cinput}\\ 0 \end{array}\right)+\left(\begin{array}{ccc} -k_{1} & 0 & 0\\ 0 & -k_{2} & 0\\ \alpha_{3,1}k_{1} & \alpha_{3,2}k_{2} & -k_{3} \end{array}\right)\left(\begin{array}{c} C_{\mathrm{AG}\;\text{Litter}}\\ C_{\text{Fine roots}}\\ \mathrm{SOC} \end{array}\right)$$

where Cinput is the input of C to each compartment. Decomposition rates in each compartment i are represented by $k_{i}$, and transfer rates from a compartment j to a compartment i are represented by $\alpha_{i,j}$. In SoilR, the model was built with the function Model_14 and fitted for the entire period between 1900 and 2022. This function considered the time vector t, which contains the point in time where the solution is sought, a vector containing the initial amounts of C in each compartment, and an object describing the atmospheric ∆^14^C and the decay rate of ^14^C. The model was fitted to the observed data using the Levenberg‐Marquart method, which tries to find the best parameter values by minimizing the difference between model predictions and observed data. The radiocarbon data from 2012 to 2019 were fitted together as a time series to better integrate the dynamics of ∆^14^C in each compartment in relation to the atmosphere. The ∆^14^C contents, the C contents, and the amount of C release for each compartment as a function of time were calculated by the functions getF14C, getC, getReleaseFlux, respectively.

We calculated the system's age, the age distribution of specific compartments and the transient time distribution using the approach developed by Metzler and Sierra (2018) by considering the vector of input I and the matrix B containing the best parameters values of decay and transfer rates for each compartment. Using the age distributions of C estimated above, we computed the ∆^14^C distribution in SOC in both the control and warming treatments using the algorithm introduced by Chanca et al. (2022). Theses ∆^14^C distributions are helpful because they can show the proportional mass distribution of carbon for different ∆^14^C values or classes for a specific year of sampling and can thus inform whether the ∆^14^C distribution in control and warming treatments differ. The algorithm, first, normalizes the time variable of the atmospheric ∆^14^C and the age distribution curves obtained previously. In the second step, both curves are divided into discrete intervals, and the final step combines the mass distribution of discrete age classes with the ∆^14^C atmospheric curve.

### 2.8 Dissolved Organic Carbon Fluxes

DOC fluxes in throughfall and deep percolation water were assessed between May 2019 and December 2021. Throughfall was collected in 15 light‐protected vessels (diameter 17 cm). All vessels were emptied fortnightly (snow‐free season) and monthly (winter) for a determination of throughfall amount. A subsample was taken from three assigned vessels for the determination of DOC and nutrient concentrations. Throughfall was immediately filtered (0.45 μm nylon mesh filter) and then frozen until analysis (see below). In autumn 2018, a pair of ceramic suction cups (24 cm length, 4.5 cm diameter) was installed at ~20–30 cm soil depth (the uppermost layer of the C horizon) at each plot. Suction of 1.5 bar was applied automatically every 6 h for 30 min to all cups during the snow‐free season. Freezing of water in tubing prevented wintertime sampling. Soil solution was collected in 1 L glass storage bottles. Soil solution was collected fortnightly, and the two solution samples from each plot were pooled to one sample for further analyses. Soil solution was immediately filtered (0.45 μm nylon mesh filter) after sampling and then frozen. Before analysis, biweekly collected samples were pooled to monthly samples, and DOC concentrations were measured with an elemental analyzer (multi N/C 2100 S Analyzer, Analytik Jena, Germany).

The mechanistic Soil‐Vegetation‐Atmosphere‐Transport Model LWFBrook90R was applied for the simulation of deep percolation from the soil profiles. The model has recently been implemented in the R environment (Schmidt‐Walter et al. 2020). The model was parameterised for each individual plot with specific soil physical characteristics, soil depth, and SOC content. Above‐ground parameters of the forest trees were set equally for all treatment plots. Time series of climate data were obtained from the site weather station, and additional parameters (windspeed, irradiance) were interpolated using nearby weather stations' data and statistical modeling (Gadermaier et al. 2024). Daily deep percolation fluxes were modeled for the whole time period where measured climate data from the weather station were available (2005–2022). DOC fluxes in throughfall and deep percolation were estimated by multiplying the sample DOC concentrations by the amount of throughfall/percolation water during the preceding inter‐sampling period.

### 2.9 Data Analysis

Raw data such as chamber CO_2_ headspace concentrations, high‐resolution soil temperature and moisture, and climate data were first processed in Microsoft Excel (CO_2_ flux calculations; daily means of soil climate and air climate data; processing of daily soil CO_2_ effluxes). Temporary malfunction of dataloggers led to several gaps in soil temperature and soil moisture recordings. However, periods in which all four data loggers in operation failed at the same time were rare (0.16% of the whole study period). During such gaps, soil temperatures were calculated using air temperature and the relationship between soil and air temperature preceding the data gap and a lag term. More often, one of the four data loggers would fail while the others continued to work. In such cases, data gaps were filled using the measured soil temperature in an adjacent plot and an adjustment term in the case that there was a slight difference in soil temperatures among those plots prior to the data gap. The data file including measured CO_2_ fluxes, modeled daily CO_2_ fluxes, manually measured soil temperature and moisture, permanently measured soil temperature, air temperature and humidity, and precipitation is available at https://doi.org/10.5061/dryad.bcc2fqzsc.

Further data processing and statistical analysis were conducted in R (R Core Team 2021). Linear mixed effects models and ANOVA functions of the *nlme* R package (Pinheiro et al. 2014) were used to test for the fixed effects of warming on soil CO_2_ efflux and soil moisture, as well as soil carbon concentration and soil carbon stocks. Blocks were used as random effects to account for repeated measurements. Data were tested for normal distribution and variance homogeneity prior to analysis. Applying a model term to account for variable variance structure among control and warming treatments improved the models significantly (Zuur et al. 2009). Separate models were applied to data obtained for the entire study period, during warming and non‐warming periods, seasons, and each year. In addition, the effects of warming, year, and their interaction on soil CO_2_ efflux were tested. Differences in fine root respiration rates were tested using a paired t‐test, with the adjacent control and warmed plots serving as pairs.

To compare the response of soil CO_2_ fluxes to warming throughout the study period and to relate these to environmental variables, the relative increase in soil CO_2_ efflux per degree of warming was calculated for each block and sampling date when the warming system was turned on:

$$\begin{array}{c} \text{Relative}\;{\mathrm{CO}}_{2}\;\text{increase}\;\mathrm{per}\;1^{{}^{\circ}}C=\\ \left(\left(R_{\text{warming}}-R_{\text{control}}\right)/\left(T_{\text{warming}}-T_{\text{control}}\right)+R_{\text{control}}\right)/R_{\text{control}}, \end{array}$$

where *R*_warming_, *R*_control_, *T*_warming_, and *T*_control_ are the measured soil CO_2_ efflux rates and soil temperatures of the control and warming plots, respectively. Only measurements where soil temperature differences between control and warming plots were > 2°C were used for this analysis (to remove dates of warming system failure). The year 2005 was warming year 1 of the three plots established in 2005. The year 2008 was warming year 1 of the three plots established in 2008. The year 2014, during which warming had been stopped, was not included in this analysis. The analysis of relative warming responses was performed for all warming years during which plot replication was *n* = 6 plots (14 years in total). Relative CO_2_ increase per degree soil warming was subsequently averaged per warming year. Annual relative CO_2_ increase per degree of warming was then correlated with average control plot soil temperatures, air temperatures, and vapor pressure deficits during corresponding periods.

## 3 Results

### 3.1 Soil CO_2_ Efflux and Root Respiration

**FIGURE 2:**
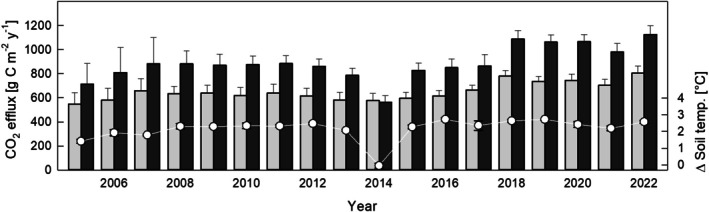
Mean annual cumulative soil CO_2_ efflux from control (gray bars) and warmed (black bars) soil. Error‐bars represent the standard error (*n* = 3 in 2005, 2006, and 2007; *n* = 6 during all other years) of the mean. Soil was warmed by 4°C during the snow‐free season. During snow‐cover and during the entire year 2014, soil warming was suspended. White circles show the mean (±SE) annual soil temperature differences between warmed and control plots.

**FIGURE 3:**
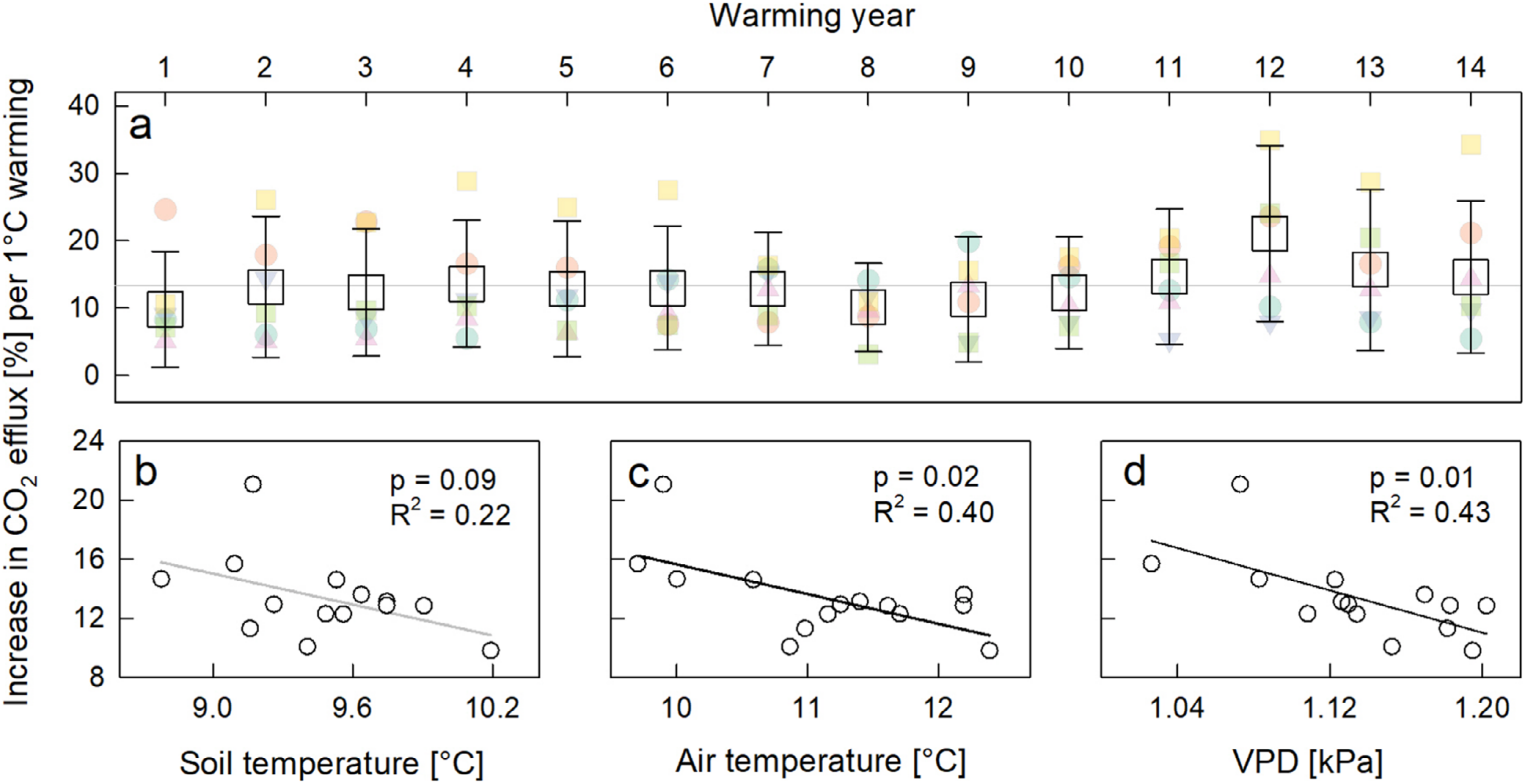
(a) Relative increase (%) of the soil CO_2_ efflux per 1°C soil warming (±SD) during 14 (out of 15) consecutive soil warming years. The normalization to 1°C warming assured interannual comparability as soil warming not always exactly reached the targeted 4°C temperature difference (Figure S1). The year 2014, during which warming was suspended, was not included in the analysis. Colored symbols show the individual responses of each of the six control/treatment blocks. The lower panel shows the relation between the mean annual CO_2_ efflux response to 1°C warming and the mean (b) soil temperature, (c) air temperature, and (d) vapor pressure deficit during the corresponding CO_2_ efflux measurements.

As a result of the warming treatment, the mean annual soil temperatures rose by 1.5°C to 2.8°C (Figure 2), depending on the number of days on which the soil was exposed to the 4°C warming treatment (*r* = 0.96, *p* < 0.001). Soil warming significantly (*F* = 317.8, *p* < 0.001) and consistently (two‐way Anova, treatment: year interaction: *p* = 0.72) increased the soil CO_2_ efflux (Figure 2, Figure S1). Annual cumulative soil CO_2_ efflux from warmed plots was between 30% and 45% higher, and the interannual variation was largely dependent on the soil warming days per year (Figure S2). In phases without soil warming, CO_2_ fluxes did not differ between treatments. During soil warming, the soil CO_2_ efflux increased by an average of 13.4% for each °C increase in soil temperature, and the responsiveness of the CO_2_ efflux to warming showed neither a declining nor an increasing trend throughout the experiment (Figure 3a). Interannually, the responsiveness of soil CO_2_ efflux to warming decreased with increasing soil and air temperatures and vapor pressure deficit (VPD) (Figure 3b–d). Cumulative CO_2_ emissions (2005–2022) from warmed soil exceeded those from non‐warmed soil by 3.95 ± 0.74 kg C m^−2^ (scaled up with a soil temperature‐dependent model) or 4.04 ± 0.76 kg C m^−2^ (scaled up by linear interpolation between flux measurements). Until the SOC stock assessment in 2019, cumulative soil CO_2_ emission caused by warming amounted to 3.03 ± 0.52 kg C m^−2^.

**FIGURE 4:**
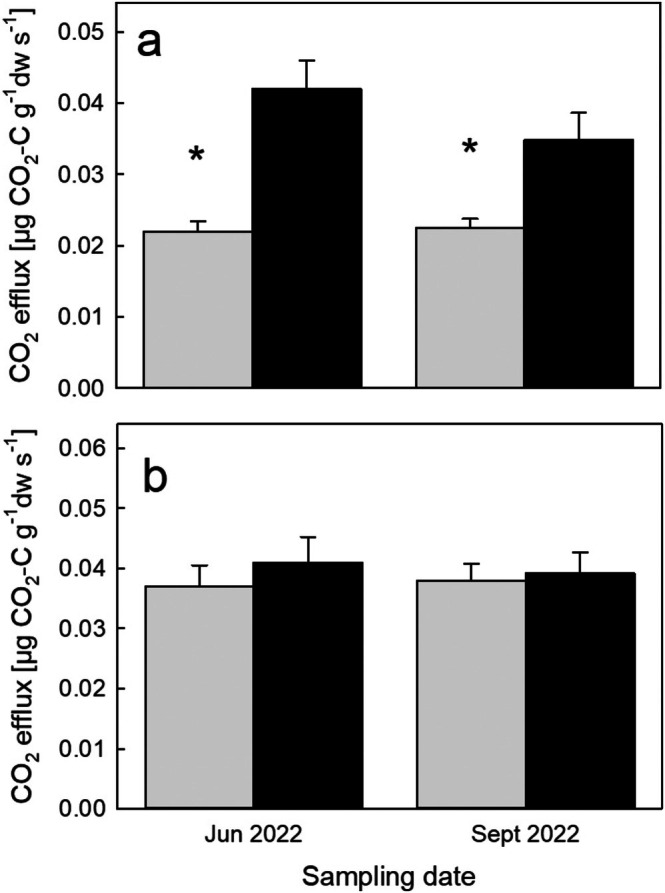
(a) Mean respiration rates (±SE, *n* = 6) of tree fine roots excavated from control plots (gray bars) and warmed (+4°C) plots (black bars). (b) Mean respiration rates of the same roots (±SE, *n* = 6) at the same temperature of 17°C. Asterisks indicate a significant difference between treatments (paired *t*‐test).

Respiration of tree fine roots (< 2 mm) excavated from warmed plots and incubated at elevated temperature (Δ + 4°C) was 50%–90% higher (*p* < 0.001) than that of roots from control plots incubated at ambient soil temperature (Figure 4a). When incubated at the same temperature, root respiration did not differ between treatments (Figure 4b), suggesting that its temperature sensitivity was not affected by long‐term soil warming.

### 3.2 DOC Fluxes

DOC input in throughfall amounted to 9.59 g C m^−2^ soil year^−1^ and 9.43 g C m^−2^ year^−1^ in 2019 and 2020, respectively. Seepage DOC leaching amounted to 10.10 ± 3.06 g C m^−2^ year^−1^ versus 12.36 ± 4.58 g C m^−2^ year^−1^ and 7.98 ± 2.32 g C m^−2^ year^−1^ versus 8.45 ± 3.10 g C m^−2^ year^−1^, in control versus warming treatments, during 2019 and 2020, respectively. Warming effects on soil DOC leaching were not statistically significant.

### 3.3 Soil Organic Carbon Concentrations and Stocks

SOC concentrations at 0–10 cm soil depth did not differ between treatments and sampling years, but in 2019, SOC concentrations were significantly lower (*p* < 0.05) in the 10–20 cm soil layer, as well as across the whole 0–20 cm warmed soil (Table 1). Higher bulk densities in warmed soil indicate that warming has compacted the topsoil. Correcting for that, the mean SOC stock was 0.9 kg C m^−2^ lower in the warmed plots, but the difference to the control plots was not significant (Table 1). SOC stocks without correction for soil compaction are given in Table S1. Mean annual input of aboveground litter was 190 g C m^−2^ at the study site between 2007 and 2019.

**TABLE 1:** Soil organic carbon concentrations and stocks in the mineral topsoil of warmed and control plots.

Year	Soil layer depth (cm)	SOC concentration (%)		Bulk density (g cm^−3^)		Corrected soil layer depth (cm)*	SOC stock (kg C m^−2^)	
		Control	Warmed	Control	Warmed		Control	Warmed
2009	0–10	11.1 (2.0)a	11.6 (1.7)a					
2019	0–10	12.3 (2.0)a	11.5 (2.4)a	0.53 (0.13)a	0.63 (0.18)a	0–8.9 (1.4)	6.3 (1.2)a	5.8 (0.5)a
2019	10–20	7.1 (1.4)a	5.7 (1.9)b	0.59 (0.11)a	0.73 (0.16)a	8.9 (1.4)–17.0 (3.1)	4.5 (1.4)a	4.1 (0.5)a
2019	0–20	9.7 (0.6)a	8.6 (0.6)b			0–17.0 (3.1)	10.8 (2.4)a	9.9 (0.6)a

*Note:* Different letters indicate statistically significant (*p* < 0.05) treatment differences (control vs. warmed).

* Layer depths were corrected for potential soil compaction (Verbrigghe et al. 2022) during long‐term soil warming.

### 3.4 Radiocarbon Signature and Age Distribution of SOC

Soil warming had no significant effect on the radiocarbon signatures of SOC; however, Δ^14^C in SOC slightly increased from 2012 to 2019 for both depths and treatments (Table S2). This slight increase in Δ^14^C in SOC differed from the decrease in Δ^14^C for litter and fine roots, which followed the temporal pattern in Δ^14^C in atmospheric CO_2_ (Figure S3). The C age distributions obtained from the three‐pool compartmental model (Figures S4 and S5) revealed that soil warming decreased the mean and median C age in the three‐pool system as well as in the SOC pool (Table 2). Changes in mean and median age due to warming were stronger for the 10–20 cm depth than for the surface 0–10 cm depth. Comparing the age distribution functions between the two treatments, we found that warming increases the proportion of young C and decreases the proportion of old C relative to the control for the entire pool system (Figure S4). For the SOC pool, warming increases the proportion of C younger than 150 years at 0–10 cm depth, while at 10–20 cm warming increases the proportion of C between 3 and 555 years old (Figure S5).

**TABLE 2:** Mean age of carbon in the SOC pool and the combined three‐pool system containing litter, fine roots, and SOC at 0–10 cm and 10–20 cm depth in the control and warming plots.

Soil depth (cm)	System age (years)		SOC age (years)	
	Control plots	Warmed plots	Control plots	Warmed plots
0–10	137 (90)	129 (83)	154 (108)	148 (104)
10–20	626 (422)	436 (297)	664 (462)	460 (321)
0–20	312 (143)	247 (130)	312 (168)	324 (154)

*Note:* The median age is given in brackets.

## 4 Discussion

### 4.1 Warming Increases the Soil CO_2_ Efflux

While DOC leaching was minor in quantity and unaffected, soil warming persistently increased the soil CO_2_ efflux throughout almost two decades. In contrast, in other long‐term soil warming experiments in forests soil respiration responses typically leveled out after few years (Bai et al. 2023; Lim et al. 2019) or started to oscillate as a matter of microbial adaptation to changing substrate availability and/or quality (Melillo et al. 2017). Our results suggest that even after 18 years of warming there is still a large SOC pool available to spur respiration rates. In our experiment, soil CO_2_ efflux rose sharply within hours after switching on the heating system in spring, and this pattern was maintained over the entire study period (Figures S1 and S6). Such a prompt response of soil CO_2_ efflux to increased soil temperature can only be explained by immediately accelerated physiological activity, that is, by stimulation of respiration, whether autotrophic and/or heterotrophic. The almost identical CO_2_ efflux from both treatments during the year without warming (2014, Figure 2) further confirms that respiration was not up‐ or downregulated at that time.

**FIGURE 5:**
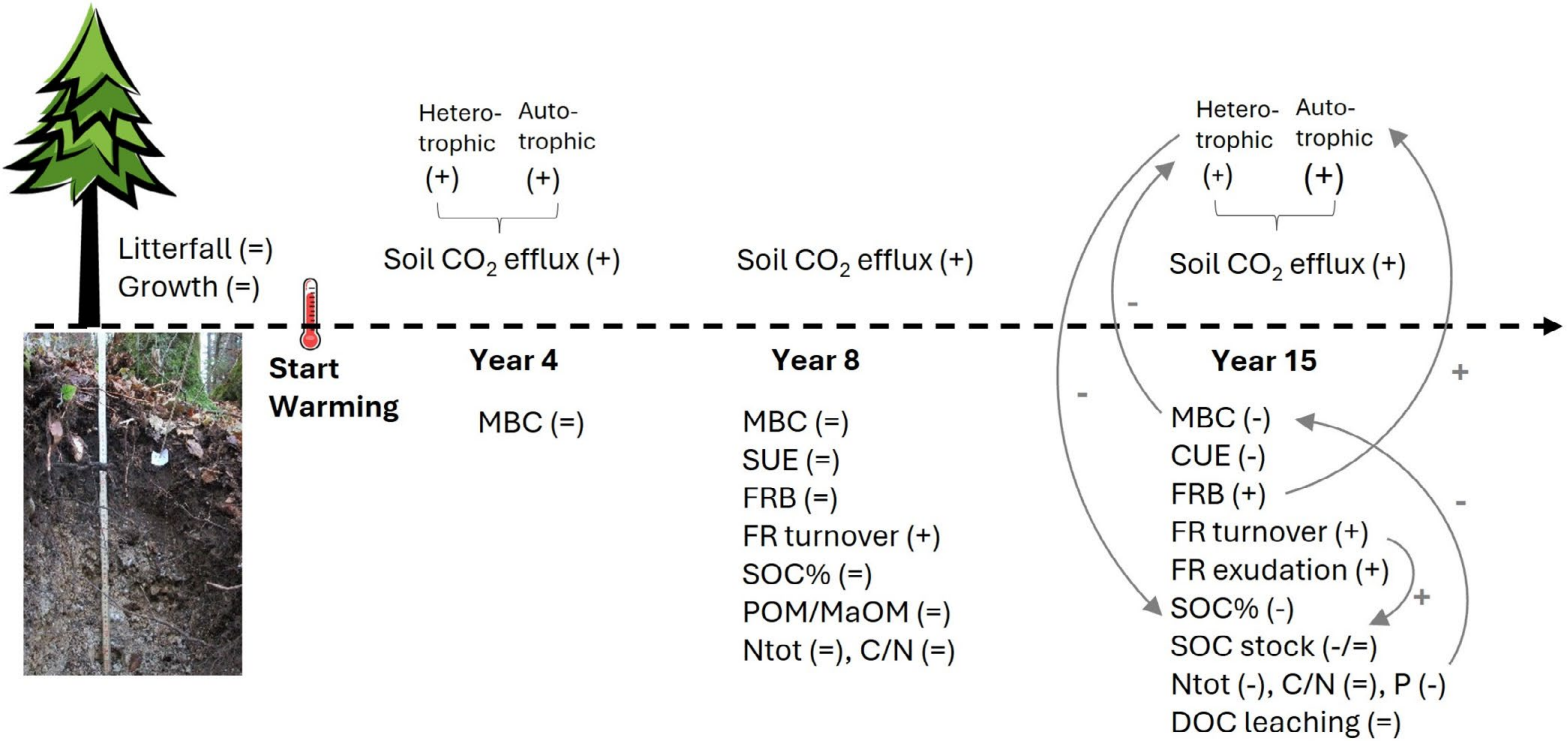
Conceptual drawing of observed responses (“+” increase, “−” decrease, “=” no change) of element fluxes, concentrations and stocks throughout the warming experiment. Gray arrows indicate potential feedback effects. Litterfall and tree growth can be considered unaffected due to the small plot size. CUE, carbon use efficiency; DOC, dissolved organic carbon; FRB, fine root biomass; MaOM, mineral associated organic matter; MBC, microbial biomass carbon; Ntot, total nitrogen concentration; P, phosphorus concentration; POM, particulate organic matter; SOC stock, organic carbon stock; SOC%, organic carbon concentration; SUE, substrate use efficiency.

Warming can stimulate both, heterotrophic and autotrophic soil respiration simultaneously (Schindlbacher et al. 2008), whereas only stimulated heterotrophic respiration can deplete the SOC pool. In the studied soil, warming increased heterotrophic soil respiration during most stages of the experiment, as also demonstrated ex situ in intact soil cores after 9 years of warming (Schindlbacher et al. 2015). The first long‐term effects on the physiology of microbial decomposers were observed after 15 years of soil warming when warming led to microbial nutrient limitation, particularly microbial phosphorus limitation (Tian, Shi, et al. 2023), which in turn reduced microbial biomass and negatively affected microbial growth and CUE (Tian, Schindlbacher, et al. 2023) (Figure 5). Though this was not yet reflected in a reduced soil CO_2_ efflux it indicates a down‐regulation of microbial (heterotrophic) respiration during the later study years (2019–2020). On the other hand, warming persistently stimulated root respiration by 50%–90% (Figure 4), suggesting that root respiration has a strong temperature sensitivity exceeding that of SOC mineralization. Acclimation of root respiration to soil warming was observed for fine roots of certain deciduous temperate tree species (Jarvi and Burton 2020; Muratore et al. 2024), but such a response was not found for fine roots of Norway spruce in our experiment (Figure 3). Combined with higher fine root biomass and an increased number of ectomycorrhizal root tips (~+80%) (Kwatcho Kengdo et al. 2022, 2023), this indicates that autotrophic soil respiration dominated the continued positive response of soil CO_2_ efflux to warming, likely even more so than at the beginning of the experiment.

While the summed response of soil CO_2_ efflux to warming remained broadly stable throughout our experiment, it still varied interannually (Figure 3). We expected that this variation was related to the occurrence of dry and wet years, as an earlier rainfall exclusion experiment at the same site demonstrated that severe soil drying offsets the stimulatory effects of soil warming (Schindlbacher et al. 2012). Soil moisture modulated effects on soil respiration were also observed in another long‐term warming experiment in a boreal forest (Liang et al. 2024). However, neither annual nor seasonal precipitation sums explained the interannual variation at our site. This indicates that soil moisture does not control the warming stimulation of soil CO_2_ efflux in the studied forest, which rarely experienced more than 2‐ to 3 weeks without precipitation, even during dry years. However, the strong negative correlation with vapor pressure deficit (Figure 3) indicates that low atmospheric humidity instead of soil drought could be a driver of interannual variation in warming induced stimulations of soil respiration, for example, via stomatal reduction of tree photosynthesis reducing belowground C allocation and autotrophic respiration.

### 4.2 Warming Increases Belowground Carbon Allocation

In our experiment, warming had no effect on aboveground litterfall, as the aboveground compartments of trees were not warmed. However, the rhizosphere showed clear responses: biomass, turnover and C exudation of fine roots increased in the warmed soil (Heinzle, Liu, et al. 2023; Kwatcho Kengdo et al. 2023, 2022), indicating greater belowground C allocation. An assessment in the middle of the experiment (year 8) already indicated an increased fine root turnover and thus increased input of root C into the soil, which was confirmed by doubling of fine root production in warmed soil during the later stage of the experiment (Kwatcho Kengdo et al. 2023) (Figure 5). The metabolomes of fine roots (Liu, Heinzle, et al. 2024) and exudation (X. Liu, pers. comm.) from individual roots was not affected by warming, but the increase in fine root biomass led to overall higher C exudation rates into the warmed soil (Heinzle, Liu, et al. 2023). Warming may also affect mycorrhizal dynamics and C flows. Ectomycorrhizal root tip number increased by about 80% in the warmed plots (Kwatcho Kengdo et al. 2022, 2023), indicating the potential for increased mycorrhizal colonization. Although we found evidence for decreased fungal biomass, mycorrhizal fungi seemed to benefit from warming when compared to saprotrophic fungi. This functional shift is supported by a change in the fungal community structure towards a greater proportion of mycorrhizal fungi (M. Ullah, pers. comm.). Quantitatively assessing mycorrhizal turnover was beyond our abilities, but the belowground C input via mycorrhiza, if accelerated like that of fine roots, could be significant. While fine root biomass and belowground C inputs increased in our experiment and in a warmed boreal forest soil (Leppälammi‐Kujansuu et al. 2014), fine root biomass significantly decreased in a temperate deciduous forest (Melillo et al. 2017) and in an alpine forest (Dawes et al. 2015) under soil warming. This emphasizes that the long‐term responses of belowground C allocation in forests can be highly site‐specific and may therefore depend strongly on ecosystem properties such as tree species composition or soil nutrient supply.

### 4.3 SOC Stocks and Radiocarbon Signatures

After 14 years of soil warming, SOC stocks were ~0.9 kg C m^−2^ lower than in control plots, but due to the heterogeneity of C contents and bulk densities among the individual plots, the effect was statistically not significant (Table 1). However, the increase in soil CO_2_ efflux and the radiocarbon age distribution patterns also suggest small SOC losses. Until the year of the SOC stock assessment, cumulative soil CO_2_ efflux caused by warming amounted to ~3.0 kg C m^−2^ and a trenching experiment during the first years suggested roughly 40% autotrophic contribution to the soil CO_2_ efflux in both control and warmed plots (Schindlbacher et al. 2009). Thus, about 1.8 kg CO_2_‐C m^−2^ would have been of heterotrophic origin, which is twice as much as the apparent SOC loss in the warmed soil. The missing portion of 0.9 kg C m^−2^ (0.064 kg m^−2^ on an annual basis) could originate from the mineralization of increased belowground C inputs to the warmed soil. This assumption aligns well with increased fine root turnover (+0.06 kg C m^−2^ year^−1^, Kwatcho Kengdo et al. 2023) and root exudation (+0.01 kg C m^−2^ year^−1^, Heinzle, Liu, et al. 2023), which sum up to roughly the C input needed to close the C budget (0.9 kg C m^−2^ divided by 14 years, giving 0.064 kg C m^−2^ year^−1^). This budget approach demonstrates that the soil CO_2_ efflux alone is a weak predictor for SOC losses by warming. Understanding the long‐term changes in SOC stocks requires quantitative measurements of in situ belowground C allocation.

The mean SOC age of 312 years (0–20 cm) in the control plots is lower compared to the average SOC age in topsoils of temperate forests (440 years) (Shi et al. 2020), suggesting relatively fast turnover of the SOC pool at our site. Calcareous soils differ from most other temperate forest soils in their low thickness, high pH value, intensive bioturbation, and markedly high SOC concentrations for mineral soils. The lower ^14^C age of SOC in warmed plots points to rejuvenation of the SOC pool at 10–20 cm depth. A substitution of older SOC by new plant‐derived C is supported by higher fine root biomass and turnover in warmed soil at 10–20 cm (Kwatcho Kengdo et al. 2023, 2022). At 0–10 cm depth, the radiocarbon age of SOC was similar in the warmed (148 years) and control (154 years) plots. In this depth, the radiocarbon age of SOC is not yet affected by increased root C input with warming. This could be related to high SOC content, relatively high proportion of old SOC with Δ^14^C signatures > 100‰ (Figure S5) and additional C input by aboveground litter compared to the 10–20 cm depth. Input of root C with Δ^14^C signatures close to 0‰ has thus little influence on the mean Δ^14^C signature and age of SOC. At the same time, the retention of new, young plant and microbial‐derived SOC (Liu, Tian, et al. 2024) is low in this carbon‐rich topsoil, mainly due to microbial respiration.

Radiocarbon investigations showed that all SOC fractions are to some extent vulnerable to rising temperatures (Vaughn and Torn 2019). This is in line with a previous study at the Achenkirch site (year 8), where the chemical composition of soil organic matter in density fractions was similarly affected by soil warming (Schnecker et al. 2016). Even with signs of SOC substitution and rejuvenation of the SOC pool at 10–20 cm depth, the ^14^C signatures suggest that the entire SOC pool responds only slowly to soil warming.

### 4.4 Forest Soils in a Warming Climate

While most forests, including tropical to boreal forests, observed short‐term increases in soil respiration in response to soil warming, we here show that soil warming can trigger a persistent long‐term increase in CO_2_ effluxes from a calcareous carbon‐rich temperate forest soil. Such long‐term effects have not been reported before in forest soils under natural conditions. Interestingly, a recent study at Harvard Forest (Knorr et al. 2024) showed a similarly sustained response of soil CO_2_ efflux but only under a combination of soil warming and N‐fertilization (50 kg N ha^−1^ year^−1^). Analogous to our observations, the increased respiratory efflux did not lead to a concomitant reduction in SOC stocks, suggesting that higher plant C inputs compensated for increased decomposition losses (although fine root biomass was suppressed in that study). In the Achenkirch soil warming experiment, N concentrations in tree fine roots (Kwatcho Kengdo et al. 2022) and diffusive N fluxes in soils (Heinzle et al. 2021) did not indicate limitations to plant N uptake in the long‐term warmed soil, probably as a matter of relatively high N deposition (~15 kg ha^−1^year^−1^) and accelerated SOM mineralization, which had made further N plant available. In the calcareous soil in Achenkirch, long‐term warming particularly affected phosphorous cycling (Shi et al. 2023; Tian, Shi, et al. 2023), which already affects microbial functioning (Liu, Tian, et al. 2024; Tian, Schindlbacher, et al. 2023) and may feed back on soil respiration and SOC under prolonged warming. Soil nutrient status also affects mycorrhizal symbionts, whose responses to warming are poorly understood, although it is likely that soil warming alters mycorrhizal growth and composition of the fungal community (Kwatcho Kengdo et al. 2022; Leppälammi‐Kujansuu et al. 2013), with potentially significant effects on belowground C allocation (Bunn et al. 2024). Accordingly, nutrient availability might be key to better understanding if and why warming adversely affects belowground C allocation in different forest ecosystems.

Importantly, the aboveground compartment is typically not heated in this and most other warming experiments in forests. Thus, in a real warmer world, C inputs into soil are likely different. If warming promotes forest growth, the plant C input into the soil may be even higher and offset any SOC mineralization losses, such as recently shown in a transplanted subtropical forest soil (Liu, Lie, et al. 2024) and as common patterns across natural temperature gradients show (Giardina et al. 2014; Ziegler et al. 2017). Overall, this study demonstrates that the response of belowground C allocation to warming is key to understanding long‐term changes in soil respiration and SOC stocks in forest ecosystems. Quantifying the contribution of roots and mycorrhizal fungi to soil respiration and SOC formation is crucial for predicting future changes in the C cycle of temperate forests with increasing temperatures. We show here that even if warming increases soil respiration in the long term, mineral SOC stocks of alpine forests on calcareous bedrock are less vulnerable to rising temperatures than previously expected.

## Author Contributions

**Andreas Schindlbacher:** conceptualization, methodology, data collection, data curation, data analysis, writing – original draft, writing – review and editing. **Werner Borken:** conceptualization, methodology, writing – original draft, writing – review and editing. **Steve Kwatcho Kengdo:** ^14^C data collection, data curation, ^14^C analysis and modeling, writing – review and editing. **Carlos A. Sierra:** ^14^C modeling and validation, writing – review and editing. **Jakob Heinzle:** data collection, writing – review and editing. **Ye Tian:** data collection, writing – review and editing. **Mathias Mayer:** data collection, writing – review and editing. **Josef Gadermaier:** data collection, writing – review and editing. **Chupei Shi:** data collection, writing – review and editing. **Caro Urbina Malo:** data collection, writing – review and editing. **Xiaofei Liu:** data collection, writing – review and editing. **Erich Inselsbacher:** data collection, writing – review and editing. **Robert Jandl:** initiation and supervision of the experiment, writing – review and editing. **Wolfgang Wanek:** conceptualization, methodology, data analysis, writing – review and editing.

## Conflicts of Interest

The authors declare no conflicts of interest.

## Supporting information


**Table S1:** Soil organic carbon concentrations and stocks in the topsoil layer of warmed and control plots without consideration of potential soil compaction.
**Table S2:** Radiocarbon signatures (∆^14^C, ‰) in aboveground litterfall, fine roots and bulk SOC. Values are means (standard deviation); *n* = 3 in 2012 and *n* = 6 in 2019, respectively.
**Figure S1:** Soil CO_2_ efflux and soil temperatures during the course of the experiment (2005–2023). Red triangles and black circles indicate mean (±SE, *n* = 3 plots from 2005 until 2008 and *n* = 6 plots from 2008 until 2022) soil CO_2_ efflux rates measured in control and warmed plots, respectively. Red (warmed) and gray (control) lines show daily modeled soil CO_2_ effluxes. The lower panel shows differences in soil temperatures at 5 cm soil depth in corresponding control and warmed plot pairs based on half hourly measurements (2005–2007 three plot pairs, 2008–2022 six plot pairs). The desired temperature difference during warming was +4°C. Warming was interrupted during snow cover and during the full year 2014.
**Figure S2:** Relationship between the duration of +4°C soil warming (days) during each study year and (a) the mean annual soil temperature difference between control and warmed plots, and (b) the annual surplus soil CO_2_ efflux from warmed plots (the annual warming effect).
**Figure S3:** Radiocarbon values for aboveground litter (AGLitter), fine roots, and SOM pools with a three‐pool compartmental system fitted to the data. A separate model was fitted to each treatment × depth combination.
**Figure S4:** Ratio of the age density function of the *three‐pool system* for warmed plots over the age density function of the control plots, for the entire 0–20 cm soil depth (top), and for the two separate soil depths (bottom). These density ratios indicate the probability of finding carbon of a given age in the warming treatment versus the control. Values above 1 indicate higher probabilities and proportions of carbon of a given age in the warming treatment relative to the control.
**Figure S5:** Ratio of the age density function of the *SOC pool* for warmed plots over the age density function of the control plots, for the entire 0–20 cm soil depth (top), and for the two separate soil depths (bottom). These density ratios indicate the probability of finding carbon of a given age in the warming treatment versus the control. Values above 1 indicate higher probabilities and proportions of carbon of a given age in the warming treatment relative to the control.
**Figure S6:** Relative difference in soil CO_2_ effluxes between control and warming plots after switching the heating system on. “warming off” indicates the difference in soil CO_2_ effluxes shortly before the heating system was turned on during spring of each study year. “warming on” indicates the difference in soil CO_2_ effluxes during the next day. The desired soil warming of +4°C was reached within 3–4 h.

## Data Availability

The data that support the findings of this study are openly available in Dryad at https://doi.org/10.5061/dryad.bcc2fqzsc.
