# Supplementary material for: Increased Belowground Carbon Allocation Reduces Soil Carbon Losses Under Long‐Term Warming
Source: Glob Chang Biol. 2025 Oct 16;31(10):e70561. doi: 10.1111/gcb.70561 (PMC12531474; doi:10.1111/gcb.70561)
Supplement: Supplementary file 1 — Table S1: Soil organic carbon concentrations and stocks in the topsoil layer of warmed and control plots without consideration of potential soil compaction. Table S2: Radiocarbon signatures (∆14C, ‰) in aboveground litterfall, fine roots and bulk SOC. Values are means (standard deviation); n = 3 in 2012 and n = 6 in 2019, respectively. Figure S1: Soil CO2 efflux and soil temperatures during the course of the experiment (2005–2023). Red triangles and black circles indicate mean (±SE, n = 3 plots from 2005 until 2008 and n = 6 plots from 2008 until 2022) soil CO2 efflux rates measured in control and warmed plots, respectively. Red (warmed) and gray (control) lines show daily modeled soil CO2 effluxes. The lower panel shows differences in soil temperatures at 5 cm soil depth in corresponding control and warmed plot pairs based on half hourly measurements (2005–2007 three plot pairs, 2008–2022 six plot pairs). The desired temperature difference during warming was +4°C. Warming was interrupted during snow cover and during the full year 2014. Figure S2: Relationship between the duration of +4°C soil warming (days) during each study year and (a) the mean annual soil temperature difference between control and warmed plots, and (b) the annual surplus soil CO2 efflux from warmed plots (the annual warming effect). Figure S3: Radiocarbon values for aboveground litter (AGLitter), fine roots, and SOM pools with a three‐pool compartmental system fitted to the data. A separate model was fitted to each treatment × depth combination. Figure S4: Ratio of the age density function of the three‐pool system for warmed plots over the age density function of the control plots, for the entire 0–20 cm soil depth (top), and for the two separate soil depths (bottom). These density ratios indicate the probability of finding carbon of a given age in the warming treatment versus the control. Values above 1 indicate higher probabilities and proportions of carbon of a given age in the warming treatm [file GCB-31-e70561-s001.docx]

**Supplement**

**Table S1.** Soil organic carbon concentrations and stocks in the topsoil layer of warmed and control plots without consideration of potential soil compaction.

| Year | Soil depth (cm) | SOC concentration (%) | | Bulk density (g cm^-3^) | | SOC stock (kg C m^-2^) | |
| --- | --- | --- | --- | --- | --- | --- | --- |
|  |  | control | warmed | control | warmed | control | warmed |
| 2009 | 0-10 | 11.1 (2.0)a | 11.6 (1.7)a |  |  |  |  |
| 2019 | 0-10 | 12.3 (2.0)a | 11.5 (2.4)a | 0.53 (0.13)a | 0.63 (0.18)a | 6.3 (1.2)a | 6.7 (1.5)a |
| 2019 | 10-20 | 7.1 (1.4)a | 5.7 (1.9)b | 0.59 (0.11)a | 0.73 (0.16)a | 4.5 (1.4)a | 3.9 (0.5)a |
| 2019 | 0-20 | 9.7 (0.6)a | 8.6 (0.6)b |  |  | 10.8 (2.4)a | 10.7 (1.5)a |

SOC stock = Bulk density x SOC concentration x soil volume. Different letters indicate statistically significant (p<0.05) treatment differences (control vs. warmed).

**Table S2:** Radiocarbon signatures (∆^14^C, ‰) in aboveground litterfall, fine roots and bulk SOC. Values are means (standard deviation); n = 3 in 2012 and n = 6 in 2019, respectively.

|  | | 2012 | | | 2019 | | |
| --- | --- | --- | --- | --- | --- | --- | --- |
| Treatment | Soil depth (cm) | Aboveground litter | Fine roots | ∆^14^C Bulk SOC | Aboveground litter | Fine roots | ∆^14^C Bulk SOC |
| Control | 0 – 10 | 46.1 (0.6) | 51.4 (6.8) | 49.8 (20.0) | 9.5 (0.1) | 35.3 (27.6) | 63.7 (13.0) |
|  | 10 - 20 | - | 70.8 (22.0) | -11.1 (16.8) | - | 30.3 (24.2) | 30.2 (20.2) |
| Warmed | 0 – 10 | 42.3 (2.0) | 44.7 (7.0) | 57.7 (10.0) | 9.5 (0.1) | 29.1 (14.1) | 58.8 (13.6) |
|  | 10 - 20 | - | 66.1 (13.4) | 13.5 (14.0) | - | 35.5 (19.7) | 17.5 (24.5) |

**Supplementary figures**


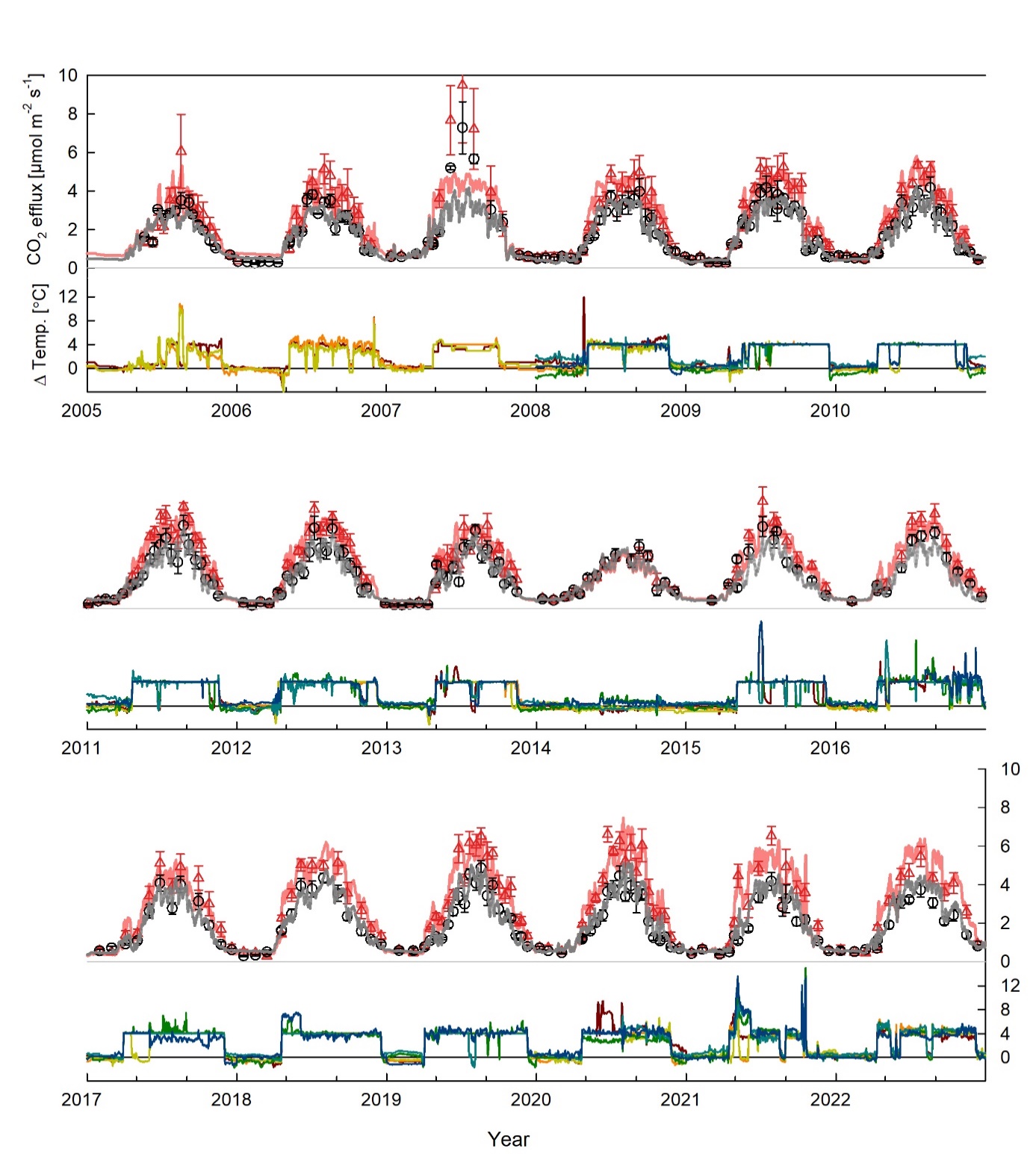


**Figure S1**: Soil CO_2_ efflux and soil temperatures during the course of the experiment (2005-2023). Red triangles and black circles indicate mean (± SE, n = 3 plots from 2005 until 2008 and n = 6 plots from 2008 until 2022) soil CO_2_ efflux rates measured in control and warmed plots, respectively. Red (warmed) and grey (control) lines show daily modelled soil CO_2_ effluxes. The lower panel shows differences in soil temperatures at 5 cm soil depth in corresponding control and warmed plot pairs based on half hourly measurements (2005-2007 three plot pairs, 2008-2022 six plot pairs). The desired temperature difference during warming was +4°C. Warming was interrupted during snow cover and during the full year 2014.


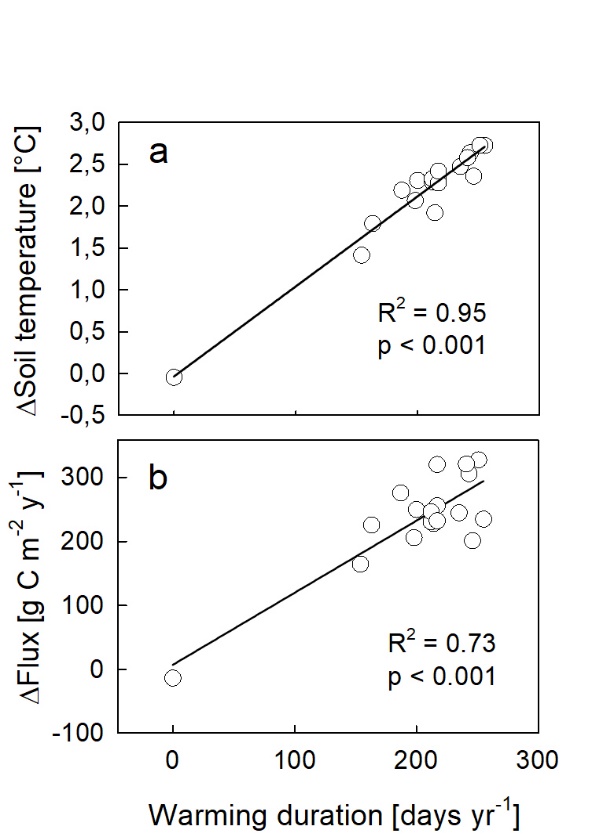


**Figure S2**: Relationship between the duration of +4°C soil warming (days) during each study year and (a) the mean annual soil temperature difference between control and warmed plots, and (b) the annual surplus soil CO_2_ efflux from warmed plots (the annual warming effect).


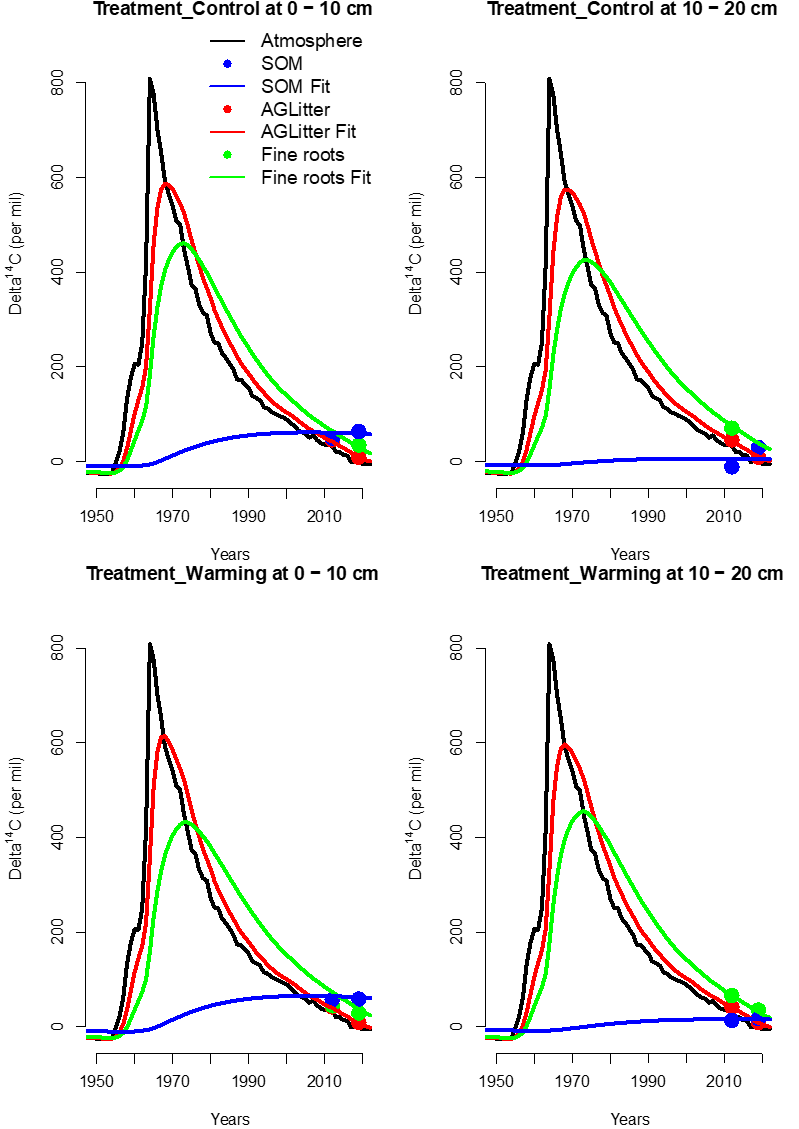


**Figure S3:** Radiocarbon values for aboveground litter (AGLitter), fine roots, and SOM pools with a three-pool compartmental system fitted to the data. A separate model was fitted to each treatment x depth combination.


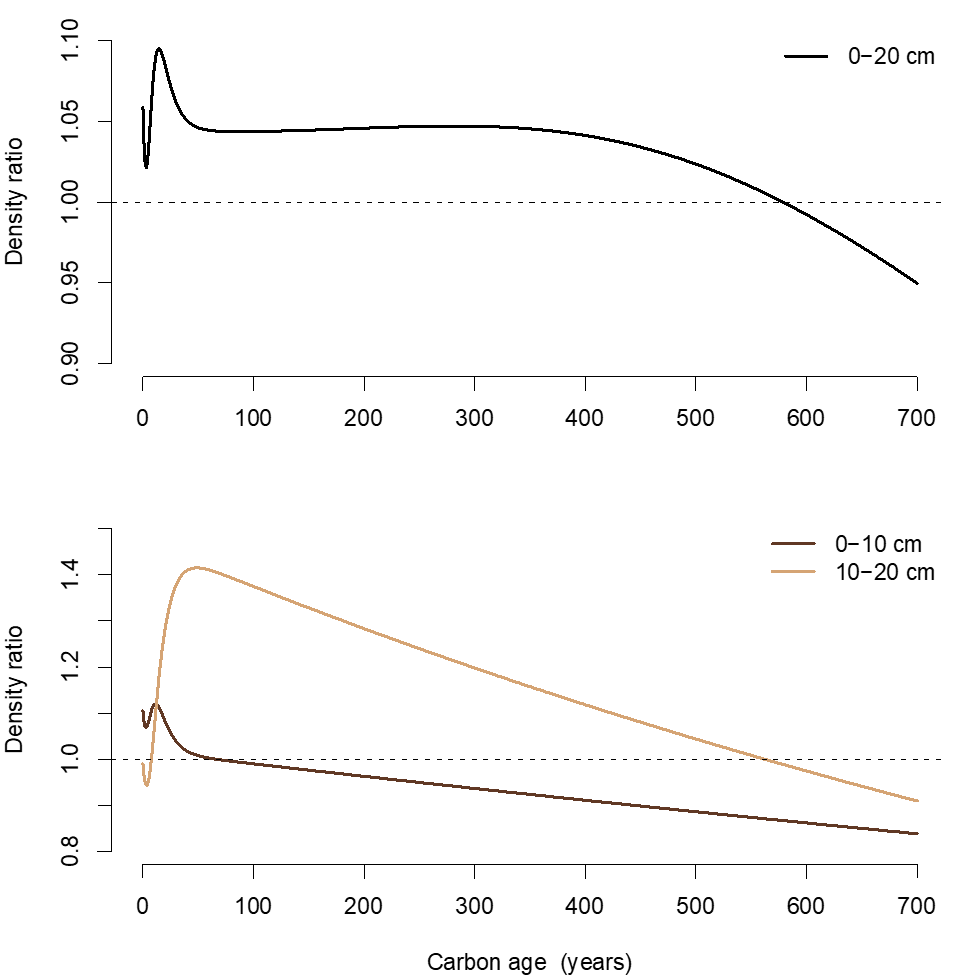


**Figure S4**: Ratio of the age density function of the *three-pool system* for warmed plots over the age density function of the control plots, for the entire 0-20 cm soil depth (top), and for the two separate soil depths (bottom). These density ratios indicate the probability of finding carbon of a given age in the warming treatment versus the control. Values above 1 indicate higher probabilities and proportions of carbon of a given age in the warming treatment relative to the control.

**Figure S5**: Ratio of the age density function of the *SOC pool* for warmed plots over the age density function of the control plots, for the entire 0-20 cm soil depth (top), and for the two separate soil depths (bottom). These density ratios indicate the probability of finding carbon of a given age in the warming treatment versus the control. Values above 1 indicate higher probabilities and proportions of carbon of a given age in the warming treatment relative to the control.


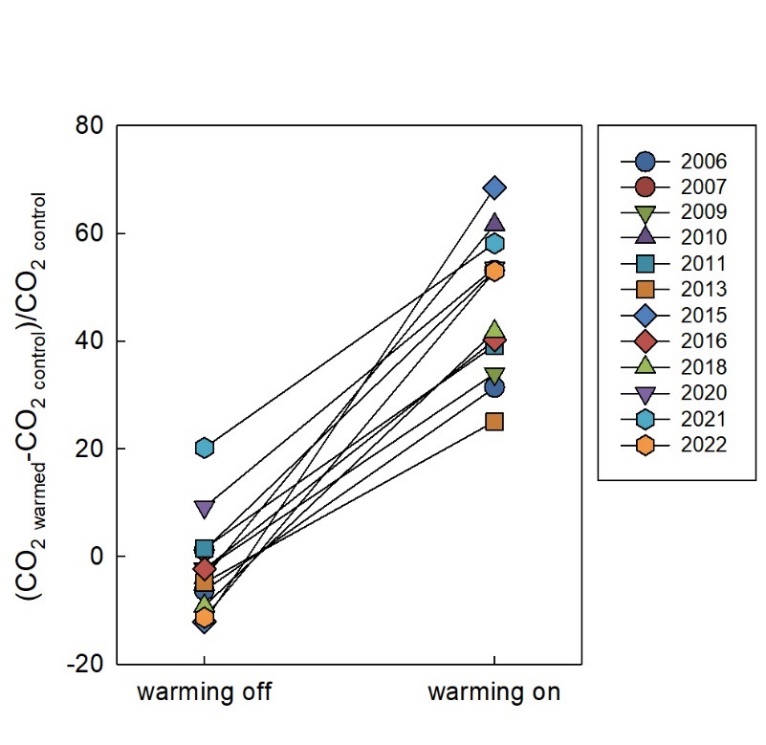


**Figure S6**: Relative difference in soil CO_2_ effluxes between control and warming plots after switching the heating system on. “warming off” indicates the difference in soil CO_2_ effluxes shortly before the heating system was turned on during spring of each study year. “warming on” indicates the difference in soil CO_2_ effluxes during the next day. The desired soil warming of +4°C was reached within 3-4 hours.
